# The Effect of Educational Programs on Hypertension Management

**Published:** 2014-09-01

**Authors:** Mohammad Ali Babaee Beigi, Mohammad Javad Zibaeenezhad, Kamran Aghasadeghi, Abutaleb Jokar, Shahnaz Shekarforoush, Hajar Khazraei

**Affiliations:** 1 Cardiovascular Research Center, Shiraz University of Medical Sciences, Shiraz, IR Iran; 2 Department of Physiology, Islamic Azad University, Arsanjan Branch, Arsanjan, Fars, IR Iran

**Keywords:** Education, Hypertension, Knowledge, Life Style

## Abstract

**Background:**

Hypertension is the main risk factor for cardiovascular diseases and stroke. Blood pressure control is a challenge for healthcare providers and the rate of blood pressure control is not more than 50% worldwide.

**Objectives:**

The present study aimed to determine the effectiveness of a short-term educational program on the level of knowledge, lifestyle changes, and blood pressure control among hypertensive patients.

**Patients and Methods:**

This quasi-experimental study was conducted on the hypertensive patients attending Shiraz Healthy Heart House. In this study, 112 patients were selected via systematic random sampling. The study data were collected using a data gathering form which consisted of baseline characteristics and measurements of blood pressure. Multivariate analyses were used to assess the relationship between education and hypertension.

**Results:**

At baseline, the scores of aware, treated, and controlled hypertensive patients were 21%, 20%, and 12%, respectively. However, these measures were increased to 92%, 95%, and 51%, respectively at the end of the study. The mean knowledge scores improved from 2.77 ± 2.7 to 7.99 ± 1.78 after 3 months (P < 0.001). Also, the mean lifestyle scores changed from 3.15 ± 1.52 to 4.53 ± 1.23 (P < 0.001).

**Conclusions:**

The results of the current study indicated that the educational programs were effective in increasing knowledge, improving self-management, and controlling detrimental lifestyle habits of the patients with hypertension.

## 1. Background

Hypertension (HTN) is the main risk factor for cardiovascular diseases and stroke. However, it is not taken seriously and is often deficiently controlled (1). Lowering the Blood Pressure (BP) reduces the associated risks. Therefore, an effective strategy for reducing HTN complications is increasing the number of patients who control BP (2, 3).

A survey of the risk factors of non-communicable diseases in Iran revealed that 25.2% and 45.5% of the adults between 25 and 64 years old had HTN and prehypertension, respectively. However, 66% of the hypertensive patients were unaware of their disorder, 75% were untreated, and 94% were not controlled. These proportions are relatively high compared to those reported in other countries (4).

Patients’ knowledge about HTN and benefits of lifestyle modifications seems to be the key to successful control of HTN (5). However, lifestyle changes are not easily achieved. Adherence to treatment increases when the patients are active (6). Therefore, well-designed educational interventions with active participation of the patients are necessary for increasing HTN knowledge, self-monitoring, and control.

## 2. Objectives

The present study aims to determine the effectiveness of a short-term educational program in BP control and adherence to healthy lifestyle.

## 3. Patients and Methods

The data were collected using a validated researcher-made questionnaire through face-to-face interviews. The participants’ demographic characteristics, including age, sex, education level, and occupation, were recorded, as well. The interview included questions about HTN knowledge (9 questions) and detrimental lifestyle behaviors (6 questions). Accordingly, one point was allocated to correct answers or behaviors and no points were considered for incorrect answers or behaviors. The total score was computed by summing up the correct answers or behaviors for each patient, ranging from 0 to 15.

Resting BP, height, weight, and BMI (kg/m^2^) were measured and cardiovascular examinations were performed for all the patients. Besides, eye examination, including visual acuity, was carried out using Snellen chart and ophtalmoscopy. In addition, ECGs were taken and interpreted by a trained resident to diagnose left ventricular hypertrophy according to Romhilt-Estes criteria. Blood samples were collected after a 12-h fasting for assessment of FBS, TG, total cholesterol, HDL, BUN, Cr, Na, and K. It should be noted that written informed consents were signed by all the participants before beginning the study.

HTN was defined according to the seventh report of the Joint National Committee on Prevention, Detection, Evaluation, and Treatment of High Blood Pressure (JNC 7). The patients were labeled as hypertensive if on the average of three measurements, Systolic Blood Pressure (SBP) was ≥ 140 mm Hg, Diastolic Blood Pressure (DBP) was ≥ 90 mm Hg, or if s/he reported current use of antihypertensive medication. The patients were considered “aware” if they gave a positive response to the question, “Have you ever been told by a doctor or another health professional that you have hypertension, also called high blood pressure?” Moreover, a patient with HTN was classified as “treated” if s/he reported taking antihypertensive medication at the time of the survey. Furthermore, a treated patient was considered “controlled” if his/her SBP was < 140 mm Hg and his/her DBP was < 90 mm Hg” (7). Overall, BP was classified as stage 1, stage 2, and severe according to the JNC 7.

### 3.1. Educational Program

At first, each patient was trained individually and face to face by a trained cardiology resident about the definitions of high BP and controlled HTN, symptoms and complications of HTN, follow up intervals, and medication adherence. Additionally, nutritional and exercise counseling was conducted by the experts at the center. A diet habit questionnaire was designed to assess the patients’ dietary patterns. The dietary recommendations included a low fat, low sodium diet with adequate consumption of fruits, vegetables, and fish. Exercise was also recommended to be done for at least 30 min/day. Then, the patients were divided into 10 groups and followed up for 3 months. Each group took part in two one-hour training sessions once a month. Class topics included definition of HTN, course of illness, symptoms, BP monitoring at home, healthy lifestyle, healthy self-management behaviors, and emphasis on the previous trainings. The patients were interviewed again after 3 months to complete the post-test questionnaire, which was exactly the same as the pre-test. Resting BP was measured again, as well.

### 3.2. Data Analysis

The SPSS statistical software, version 16 (SPSS, Inc. Chicago, IL) was used to compute the frequencies and means of the patients’ demographic characteristics and their responses to the knowledge and lifestyle behaviors test. McNemar’s test was used to analyze the categorical data. In addition, paired sample t-test was employed to analyze any changes in the mean scores of knowledge and behaviors at the end of the study. P value < 0.05 was considered as statistically significant.

## 4. Results

The present study was conducted on 100 hypertensive patients. A total of 12 patients, who failed to return for follow up, were excluded from the study. Among the study participants, 65% were male. In addition, approximately two third of the patients had below high school degrees. At baseline, 21% of the hypertensive patients were aware of their high BP, 20% of the aware patients were treated, and only 12% of the treated ones were controlled. All these variables significantly improved at the end of the study (Table 1). Moreover, the percentage of the patients taking medication during the 3-month period increased from 20 to 95.

**Table1.** Percentage of Aware, Treated, and Controlled Hypertensive Patients before and after the Intervention

Variable	Before (n = 100)	After (n = 100)	P value
**Aware, n (%)**	21 (21.00)	92 (92.00)	< 0.001
**Aware, taking medication, n (%)**	20 (20.00)	95 (95.00)	< 0.001
**Aware, taking medication, controlled** ^**a**^ **, n (%)**	12 (12.00)	51 (51.00)	< 0.001

^a^Measured SBP lower than 140 mm Hg and measured DBP lower than 90 mm Hg

According to Table 2, almost 99% of the hypertensive patients had other concomitant risk factors, the most common of which being overweight or obesity.

**Table 2.** Frequency of the Concomitant Risk Factors of Coronary Artery Disease

Variable	Number	Percent
**BMI ≥ 25**	60	60.00
**Abnormal GTT**	22	22.00
**Overt diabetes**	32	32.00
**Dyslipidemia (LDL > 130)**	22	22.00
**Renal dysfunction (GFR < 60)**	19	19.00
**Left ventricular hypertrophy**	12	12.00
**Retinopathy**	41	41.00
**Smoking**	27	27.00
**Family history of early heart disease**	26	26.00

At baseline, 11 - 90% of the responses to the questionnaires were correct (Table 3). Accordingly, the majority of the participants (90%) knew about the range of a blood pressure reading. However, a low percentage of the hypertensive patients were knowledgeable about the meaning of high BP and controlled HTN. Besides, 32% of the patients knew that HTN increases the risk of stroke, heart attack, heart failure, and kidney disease and only 24% believed that people can help lower their high BP. However, the patients had less information about more specific questions on BP. The patients’ mean scores of knowledge improved from 2.77 ± 2.7 before the intervention to 7.99 ± 1.78 after 3 months (P < 0.001).

**Table 3.** Percentage of Correct Responses to Hypertension Knowledge Questions before and after the Intervention

Variable (habits)	Before, n(%)	After, n(%)	P value
**Smoking**	27 (27.00)	25 (25.00)	0.500
**Physical inactivity**	84 (84.00)	26 (26.00)	< 0.001
**Excessive salt intake** ^**a**^	40 (40.00)	5 (5.00)	0.030
**Inadequate use of vegetables and fruit**	61 (60.00)	35 (35.00)	< 0.001
**Inadequate use of fish**	63 (63.00)	56 (56.00)	0.016
**Use of saturated fat**	4 (4.00)	1 (1.00)	0.250

^a^ Excessive salt use was considered to be ≥ 5 g/day (7)

Table 4 displays self-reported detrimental lifestyle behaviors at baseline and 3 months after the educational program. As the table depicts, the number of participants with physical inactivity, excessive salt use, and inadequate use of vegetables, fruits, and fish was significantly decreased after the intervention. The patients’ mean scores of lifestyle changed from 3.15 ± 1.52 at baseline to 4.53 ± 1.23 after three months.

**Table 4.** Percentage of Hypertensive Patients with Detrimental Lifestyle Behaviors before and after the Intervention

Variable	Before, n(%)	After, n(%)	P value
**What is the meaning of high blood pressure?**	31 (31.00)	97 (97.00)	< 0.001
**What is the meaning of controlled hypertension?**	21 (21.00)	92 (92.00)	< 0.001
**Is hypertension a chronic condition?**	39 (39.00)	85 (85.00)	< 0.001
**Do you know the two numbers reported for blood pressure?**	90 (90.00)	96 (96.00)	0.030
**What are the complications of high blood pressure?**	32 (32.00)	88 (88.00)	< 0.001
**What are the symptoms of high blood pressure?**	15 (15.00)	89 (89.00)	< 0.001
**Can you do things to lower blood pressure?**	24 (24.00)	89 (89.00)	< 0.001
**What are the follow up intervals?**	11 (11.00)	81 (81.00)	< 0.001
**What is the blood pressure measurement technique?**	13 (13.00)	97 (97.00)	< 0.001

## 5. Discussion

It has been reported that a fall of 10 - 20 mmHg in systolic pressure maintained for 5 years could reduce the risks of myocardial infarction by 25% and that of stroke by40% (8). However, BP control is a challenge for healthcare providers and the rate of BP control worldwide is on average not more than 50% and may even be as low as 8.1%. Wu Y et al. conducted a study on a group of hypertensive patients in Singapore and showed that although HTN treatment was high, its awareness and control were low (9). Similarly, in spite of the high prevalence of HTN in China, the percentage of hypertensive patients who were aware, treated, and controlled was very low (11).

The present study was a well-organized educational model which involved the patients in their own health care. This study was conducted in order to determine the status of HTN awareness, treatment, and control in the Iranian population and to evaluate the effect of a short-term educational program on the above-mentioned parameters. According to the study findings, the rate of HTN awareness (21% of those having HTN), treatment (20% of those aware of HTN), and control (12% of the hypertensive patients on treatment) was low in our population. HTN control was even less than that reported in other studies (12). However, this measure was significantly increased from 12% to 51% after the educational intervention, demonstrating the beneficial effects of education on the triad of patient’s awareness, lifestyle changes, and adherence to medications. Patient’s education, self monitoring of BP, and regular follow up were also revealed to be effective healthcare measures for controlling HTN.

Patient’s involvement in self-monitoring and management, together with continuous follow up has also been recommended by others (13, 14). Similarly, Wang YR et al. emphasized that the most important points for BP control were lifestyle modifications, home BP monitoring, reinforcement of healthy behaviors, and continuous follow up (15).

In spite of the increasing emphasis on drug therapy, lifestyle modification is an important part of BP control (16, 17). It has been found that the patients who adhered to medication and lifestyle regimens had better health outcomes (18).

Because few hypertensive patients receive guidance on changing their lifestyles, healthcare professionals should further encourage the hypertensive patients regarding lifestyle habits (1).

Moreover, Wai Chiu et al. demonstrated that follow-up calls after nursing clinic consultation were effective in improvement of the patients’ adherence to a healthy lifestyle and their BP control (9). In the present study, the proportion of physically inactive individuals, excessive salt users, and those with inadequate intake of vegetables, fruits, and fish was significantly decreased after 3 months as indicated by improvement in the patients’ life style scores. This strongly suggests the effectiveness of our approach in transferring information regarding life style changes for BP control.

One of the strong points of this study was a matched-pair analysis before and after the measurements on the same patient, because it helps match the unmeasured variables.

The present study had some limitations, with small sample size and short follow-up period being the most important ones. Further studies with larger sample sizes and longer follow-ups are therefore recommended to be conducted on the issue. Another limitation was lack of a special emphasis on smoking cessation in the educational program offered to our patients. The importance of smoking avoidance, as one of the strongest predictors of cardiovascular health and survival, has been shown in several studies (19, 20). Recently, it has been expressed that “smokers who adopted other healthy behaviors still had lower survival rates than sedentary and obese nonsmokers” (22). Unfortunately, this habit does not change easily (21) and only 2% of smokers quit smoking at the end of counseling. Thus, healthcare providers should insist more on smoking avoidance.

In conclusion, educational interventions have significantly desirable effects on lifestyle modification and BP control. Therefore, they should become an integral part of management of the patients with HTN. On the other hand, HTN control in our hypertensive population was found to be less than that reported in many other countries. Thus, a public educational program for promoting HTN awareness and lifestyle modification is an urgent need.
